# Mitochondria-Targeted Drug Delivery

**DOI:** 10.3390/pharmaceutics14010178

**Published:** 2022-01-13

**Authors:** Joanna Kopecka

**Affiliations:** Department of Oncology, University of Torino, Via Santena 5/bis, 10126 Torino, Italy; joanna.kopecka@unito.it

Mitochondria, organelles surrounded by a double membrane and with their own small genome, are the cells’ energy centres. Besides the production of ATP through cellular respiration, mitochondria play a pivotal role in other aspects of the life and death of a cell: heat production, programmed cell death, regulation of metabolic activity, immunity, and calcium homeostasis. A number of diseases are associated with mitochondrial dysfunction, including cardiovascular, neurological, inflammatory, metabolic disorders and cancer. Mitochondria therefore represent an important therapeutic target, and it is not surprising that a number of different treatment strategies have emerged. Approaches targeting mitochondria can be split into two opposite categories: drugs that restore mitochondrial function and drugs that trigger mitochondria-mediated cell death. Targeted drug delivery to obtain a selective accumulation of drug molecules in mitochondria is complex and involves methods such as direct drug modification or encapsulation into nanocarriers.

This Special Issue’s goal is to present the current state of the art in the field. We invite reviews or original articles on mitochondria as a promising target for treating a wide range of human diseases and pathological conditions, as well as articles on recent approaches for mitochondria-targeted drug delivery.

In this Special Issue, 76 authors representing 71 affiliations from 7 countries over 3 continents have made 12 contributions, and it is my great privilege and pleasure to introduce this collective work which summarizes important insights in this field of research.

As mentioned above, mitochondria contain DNA and therefore are involved in genetic disorders reviewed in this Special Issue by Jang et al. Jang et al. showed relevant core genetic components involved in mitochondria linked diseases and recent genetic approaches to alleviate and/or reverse negative effects of the core component mutations on the physiology and functions of mitochondria [1]. In particular mutations in the mitochondrial genome or in the nuclear genes encoding for proteins involved in oxidative phosphorylation (OXPHOS) may cause primary mitochondrial diseases (PMD). A group of severe often inherited genetic disorders that now are under intensive investigation aimed to find the best experimental treatments. The therapeutic approaches to treat PDM was reviewed by Bottani et al. presenting both more general approaches and precision medicine strategies [2]. Besides, genetic disorders mitochondria play also a key role in cardiovascular disfunctions as nicely summed up by Forini et al. In this interesting review, authors discuss advance in nanotechnology research aimed to improve pharmacokinetic and biocompatibility in mitochondria-targeted drug delivery systems to contrast cardiovascular diseases [3]. In line with importance of mitochondria in cardiovascular pathologies, review of Farooq et al. describes tissue condition after the stroke where sufficient mitochondrial activity is necessary for cell survival. Lacking mitochondria in damaged tissues can be obtained from astrocytes in order to rescue them, however without the capacity to completely repair damaged tissues. Farooq et al. investigate current literature about mitochondrial transfer pathway as a target of future therapeutic strategies with an emphasis on stem cells as a source of healthy mitochondria [4]. Tissue damage and mitochondria in the contest of inflammation due to the response to lipopolysaccharide (LPS) present in outer membrane of Gram-negative bacteria is an object of Fock and Parnova’s review. The authors describe the newest finding in the field of protection against LPS induced inflammatory response. LPS causes oxidative stress and mitochondrial dysfunction therefore application of different mitochondria-targeted antioxidants have beneficial influence on the cells and protects cells and tissues from damage [5]. Mitochondria are also important in cancer. Alternated tricarboxylic acid cycle leads to production of oncometabolites increasing cancer aggressiveness and drug resistance as reviewed by Godel et al. In addition to detailed description of oncometabolites role in response to anticancer therapy, authors of this review describe the state of the art in pharmacological strategies targeting oncometabolites production in order to improve the efficacy of cancer treatment [6]. Targeting mitochondria often aims to enhance apoptosis of cancer cells. For example, Mollinedo and Gajate reviewed the involvement of cholesterol transport and cholesterol-rich lipid rafts in the interactions between the organelles as well as in the role of mitochondria in the regulation of apoptosis in cancer cells and cancer therapy. In particular they concentrate their effort on the role of alkylphospholipid analogs such as ether lipid edelfosine that induces the redistribution of lipid rafts from the plasma membrane to the mitochondria, reorganization of raft-located mitochondrial proteins that critically modulate cell death or survival [7]. Targeting mitochondria often aims to inhibit cancer growth. Indeed, Lee et al. have showed that blocking of oxoglutarate carrier decreased transport of NADH from cytosol to mitochondria, resulting in efficient inhibition of melanoma growth in vitro and in vivo due to suppression of mitochondrial activity and reduction of ATP production [8]. Interestingly, efficacy of drugs targeting mitochondria depends on cell metabolism as demonstrated by Zhang et al. They showed that distinctive mitochondrial metabolism in melanoma cells characterized by different invasiveness influences cytotoxic effects of plumbagin (PLB). PLB anticancer effects are mediated partially by modulation of mitochondrial electron transport and induction of reactive oxygen species. Indeed, PLB displays stronger cytotoxic effects on A375 cells, which exhibit lower respiratory function than SK-MEL-28 cells with higher respiratory function. At contrary to SK-MEL-28, A375 cells after the treatment with PLB have decreased mitochondrial OXPHOS and ATP production with elevated mitochondrial membrane potential [9]. Similar results were obtained by Zavadskis et al. using human amniotic membrane mesenchymal stromal cells (hAMSCs), human bone marrow mesenchymal stromal cells (hBMSCs) and MG-63 cells. They have showed that cytotoxic effect of diphenyleneiodonium (DPI) depends on type of the metabolism used by the cells. Thus, in cell types prevalently using glycolysis, DPI predominantly interacts with nicotinamide adenine dinucleotide phosphate oxidase, while the mitochondria remain unaffected. In contrast, in cells with aerobic energy metabolism, the mitochondria become an additional target for DPI. As a result, cells relying more on aerobic metabolism such as MG-63 or osteoblast-like cells are more sensitive to the toxic effects of DPI, while cells predominantly living from glycolysis, such as hAMSCs, are more resistant to the toxic effects of DPI suggesting that undifferentiated cells rather than differentiated parenchymal cells should be considered as potential targets for DPI [10]. To effectively target mitochondria drugs need to be delivered specifically and although some drugs may accumulate prevalently in mitochondria due to their physicochemical nature, often a targeting vehicle is needed. Ripoll et al, have showed promising new carrier to target mitochondria based on nonprotonable, noncharged thiophene ring. Authors used this construction to prepare chimeric drug deliver using the carrier and pyruvate dehydrogenase kinase inhibitor dichloroacetate demonstrating utility of thiophene moiety as a noncharged carrier for targeting mitochondria with metabolism-targeting drugs [11]. Alternatively, the drugs maybe designed specifically. For instance, Burdukiewicz et al. have showed a robust computational tool, CancerGram helping researchers to find appropriate anticancer peptides to target mitochondria in the cancer cells [12].

In conclusion, this Special Issue underlines importance of mitochondria in pathophysiological situation such as: genetic disorders [1,2], cardiovascular diseases [3,4], inflammation [5] and cancer [6,7,8,9,10]. Indicates new tools to selective target mitochondria in order to: rescue aberrant cell phenotype, change cell metabolism or induces mitochondria dependent cell death [1,2,3,4,5,6,7,8,9,10,11,12].
